# Thymoma-Related Chest Pain and Dyspnea in a Middle-Aged Caucasian Female With Myasthenia Gravis and Good's Syndrome: A Case Report

**DOI:** 10.7759/cureus.68027

**Published:** 2024-08-28

**Authors:** Ali Z Ansari, Teja Koi, Sean Lief, Srihita Patibandla, Nilay Bhatt, Azouba Gulraiz, Muhammad Bilal, Rashad Ali

**Affiliations:** 1 Department of Pathology, William Carey University College of Osteopathic Medicine, Hattiesburg, USA; 2 Department of Internal Medicine, William Carey University College of Osteopathic Medicine, Hattiesburg, USA; 3 Department of Internal Medicine, Trinity Health Grand Rapids, Grand Rapids, USA; 4 Department of Internal Medicine, HCA Houston Healthcare Clear Lake, Webster, USA; 5 Department of Internal Medicine, Poplar Bluff Regional Medical Center, Poplar Bluff, USA; 6 Department of Internal Medicine, Merit Health Wesley, Hattiesburg, USA; 7 Department of Obstetrics and Gynecology, South Central Regional Medical Center, Laurel, USA

**Keywords:** apical pneumothorax, left-sided pleural effusion, hyperaeration, lung atelectasis, progressive dyspnea, complete surgical resection, anterior mediastinal mass, myasthenia gravis, non-cardiogenic chest pain, thymoma

## Abstract

Thymomas are rare tumors originating from thymic tissue, often associated with various paraneoplastic syndromes that can pose significant clinical management challenges. Myasthenia gravis, one of the most common paraneoplastic syndromes linked to thymomas, is characterized by autoantibodies targeting the neuromuscular junction, leading to muscle weakness exacerbated by repetitive use. Good's syndrome, an adult-onset immunodeficiency associated with thymomas, results in hypogammaglobulinemia and susceptibility to opportunistic infections, which can be life-threatening. We present the case of a 57-year-old Caucasian female with no prior medical history, who presented with a three-month history of progressive chest pain, dyspnea, and muscle weakness. A computed tomography (CT) scan of the chest revealed an anterior mediastinal soft tissue mass. Upon admission, a diagnostic workup, including serum anti-acetylcholine receptor antibodies and electromyography, confirmed the diagnosis of myasthenia gravis. Immune studies revealed hypogammaglobulinemia, consistent with Good's syndrome. The patient underwent complete surgical resection of the thymoma and received intravenous immunoglobulin (IVIG) therapy. This case report highlights the rarity and clinical significance of concurrent myasthenia gravis and Good’s syndrome as paraneoplastic manifestations secondary to thymoma. Given the incidence of thymoma-associated paraneoplastic syndromes, early recognition and intervention are crucial for optimal outcomes. Future research may further elucidate the mechanisms underlying these associations, guiding improved management strategies.

## Introduction

Thymomas are rare tumors originating from thymic tissue, with an estimated incidence ranging from 0.13 to 0.32 cases per 100,000 individuals. They constitute a small percentage of all malignancies and are not strongly associated with common carcinogenic factors such as tobacco, alcohol, or early childhood radiation exposure. Epidemiological data suggest that the incidence of thymomas varies by race, with the Caucasian race having the lowest incidence at approximately 0.13 per 100,000, followed by the Black race at 0.20 per 100,000, and other racial groups at 0.29 per 100,000. The peak incidence of thymoma diagnosis occurs in middle age, typically between 45 and 55 years old [1].

Myasthenia gravis is a common paraneoplastic syndrome associated with thymomas, characterized by the production of autoantibodies that attack the neuromuscular junction, leading to muscle weakness that worsens with repetitive use. It is important to consider thymic malignancy in the differential diagnoses of patients presenting with symptoms similar to myasthenia gravis [2]. The global incidence of acetylcholine receptor antibody-positive myasthenia gravis varies, ranging from approximately 4 to 18 cases per million person-years. Myasthenia gravis can lead to myasthenic crisis, which has an in-hospital mortality rate of 2.2% to 4.7% [3]. When myasthenia gravis occurs as a paraneoplastic syndrome secondary to thymoma, treating the thymoma may alleviate myasthenia gravis symptoms. However, in cases of acute myasthenic crisis, it is crucial to stabilize neurological symptoms before performing a thymectomy to prevent respiratory failure [2].

Good’s syndrome is a rare condition characterized by the coexistence of thymoma and hypogammaglobulinemia, leading to immunodeficiency [4,5]. Patients with Good’s syndrome often experience recurrent bacterial sinopulmonary infections, particularly with encapsulated organisms such as *Haemophilus influenzae* and *Streptococcus pneumoniae* [4]. The incidence of Good's syndrome is estimated to be around 0.15 per 100,000, highlighting its rarity [5]. Hematological manifestations of Good's syndrome commonly include anemia, present in over 50% of cases, and leukopenia, affecting approximately 55% of patients. Unlike myasthenia gravis, surgical removal of the thymoma does not resolve the immunodeficiency associated with Good's syndrome; patients typically require lifelong immunoglobulin replacement therapy [2,4]. Thymomas associated with Good's syndrome can also present with symptoms related to the mass effect, such as respiratory issues or superior vena cava compression, due to the tumor's location in the anterior mediastinum [1,6].

## Case presentation

A 57-year-old Caucasian female presented to the emergency department (ED) with a three-month history of progressively worsening chest pain, shortness of breath, and muscle weakness. She had not seen a physician in over a decade and was uncertain about her medical history. The patient reported no regular medication use. Initially, she rated her chest pain as 1/10 on the pain scale, but it had since increased to 6/10. She described the pain as dull and achy, localized to the upper chest. She also mentioned occasionally coughing up greenish-yellow sputum over the past three months. The patient denied a history of smoking, tobacco, or alcohol use and had briefly taken aspirin, which she discontinued due to a lack of pain relief. She also reported unintentional weight loss and significant fatigue over the past few months, with no changes in diet or physical activity. On physical examination, her vital signs were stable, and apart from muscle weakness, the examination was unremarkable. Laboratory tests, including a complete blood count and metabolic panel, revealed leukopenia, elevated red blood cells, hemoglobin, and red cell distribution width, as well as hypokalemia, hypercapnia, and elevated levels of blood urea nitrogen (BUN) and total bilirubin (Table 1).

**Table 1:** Laboratory findings indicating leukopenia, elevated red blood cells, hemoglobin, and red cell distribution width, along with hypokalemia, hypercapnia, and elevated levels of blood urea nitrogen and total bilirubin

Test	Observed Value	Reference Range
White blood cells	2.7 x 10^3^/μL	4.0-11.0 x 10^3^/μL
Red blood cells	5.2 x 10^6^/μL	4.0-5.0 x 10^6^/μL
Hemoglobin	15.4 g/dL	12.1-15.1 g/dL
Hematocrit	45%	42%-52%
Mean corpuscular hemoglobin	30 pg/cell	27-31 pg/cell
Mean corpuscular hemoglobin concentration	34 g/dL	33-36 g/dL
Mean corpuscular volume	82 fL	80–100 fL
Platelet count	207 x 10^9^/L	150-450 x 10^9^/L
Mean platelet volume	10 fL	8-12 fL
Red cell distribution width	16%	12%-15%
Sodium	142 mmol/L	135-147 mmol/L
Potassium	3.3 mmol/L	3.5-5.0 mmol/L
Chloride	104 mmol/L	96-106 mmol/L
Carbon dioxide	34 mmol/L	23-29 mmol/L
Blood urea nitrogen	13 mmol/L	2.1-8.5 mmol/L
Creatinine	0.9 mg/dL	0.7-1.3 mg/dL
Glucose	73 mg/dL	70-100 mg/dL
Calcium	9.1 mg/dL	8.5-10.2 mg/dL
Albumin	3.6 g/dL	3.5-5.5 g/dL
Alkaline phosphatase	112 U/L	44-147 U/L
Alanine aminotransferase	21 U/L	7-56 U/L
Aspartate aminotransferase	24 U/L	5-40 U/L
Total bilirubin	1.3 mg/dL	0.3-1.0 mg/dL
Total protein	8.1 g/dL	6.0-8.3 g/dL
Globulin	3.0 g/dL	2.0-3.5 g/dL
Lipase	102 U/L	10-140 U/L

**Figure 1:**
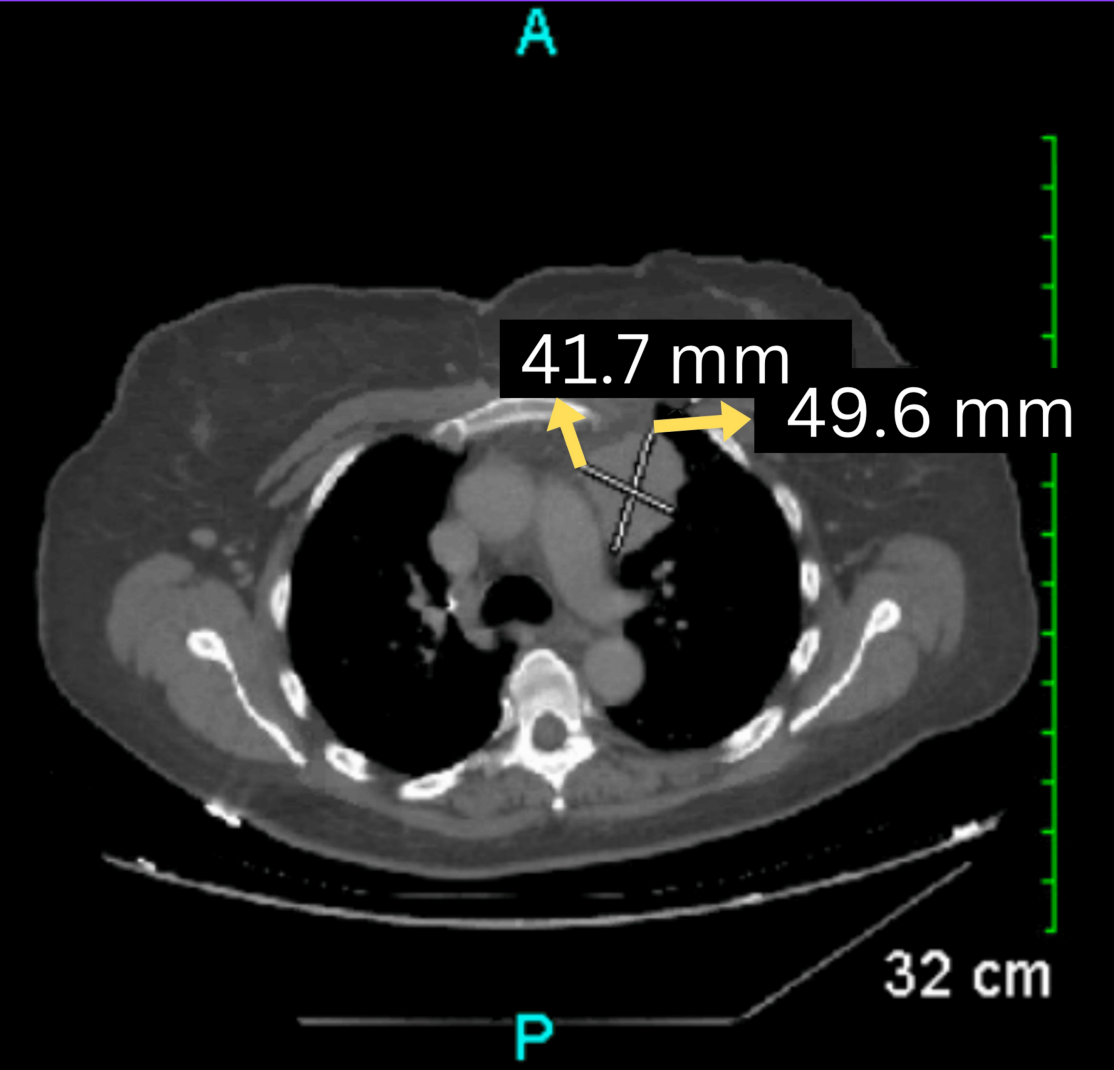
CT scan of the chest revealing an anterior mediastinal soft tissue mass measuring 5.0 x 4.2 cm (yellow arrows), located just anterolateral to the main pulmonary artery and aortic arch. No additional mediastinal or hilar masses were identified. A hiatal hernia is also noted. CT: computed tomography

A chest X-ray revealed a soft tissue mass in the anterior mediastinum. To further characterize the mass, a computed tomography (CT) scan of the chest was subsequently performed (Figure 1). Based on the clinical presentation, thymoma with possible myasthenia gravis was highly suspected. A diagnostic workup was performed, including testing for serum anti-acetylcholine receptor (anti-AChR) antibodies, which were positive, confirming the diagnosis of myasthenia gravis. Electromyography (EMG) further demonstrated a decremental response on repetitive nerve stimulation, consistent with the diagnosis.

Given the laboratory findings of leukopenia, chronic shortness of breath, and signs of a potential infection, there was concern for Good's syndrome. Immunological studies were conducted, revealing hypogammaglobulinemia, with a markedly decreased immunoglobulin G (IgG) level of 212 mg/dL (Table 2). To further investigate the underlying cause, a bone marrow biopsy was performed, which showed a reduced pre-B cell lineage, confirming the diagnosis of Good's syndrome.

**Table 2:** Immunological studies indicating hypogammaglobulinemia, characterized by a significantly reduced IgG level IgG: immunoglobulin G; IgA: immunoglobulin A; IgM: immunoglobulin M

Test	Observed Value	Reference Range
Baseline IgG	212 mg/dL	700-1,600 mg/dL
Baseline IgA	196 mg/dL	90-386 mg/dL
Baseline IgM	91 mg/dL	20-172 mg/dL

**Figure 2:**
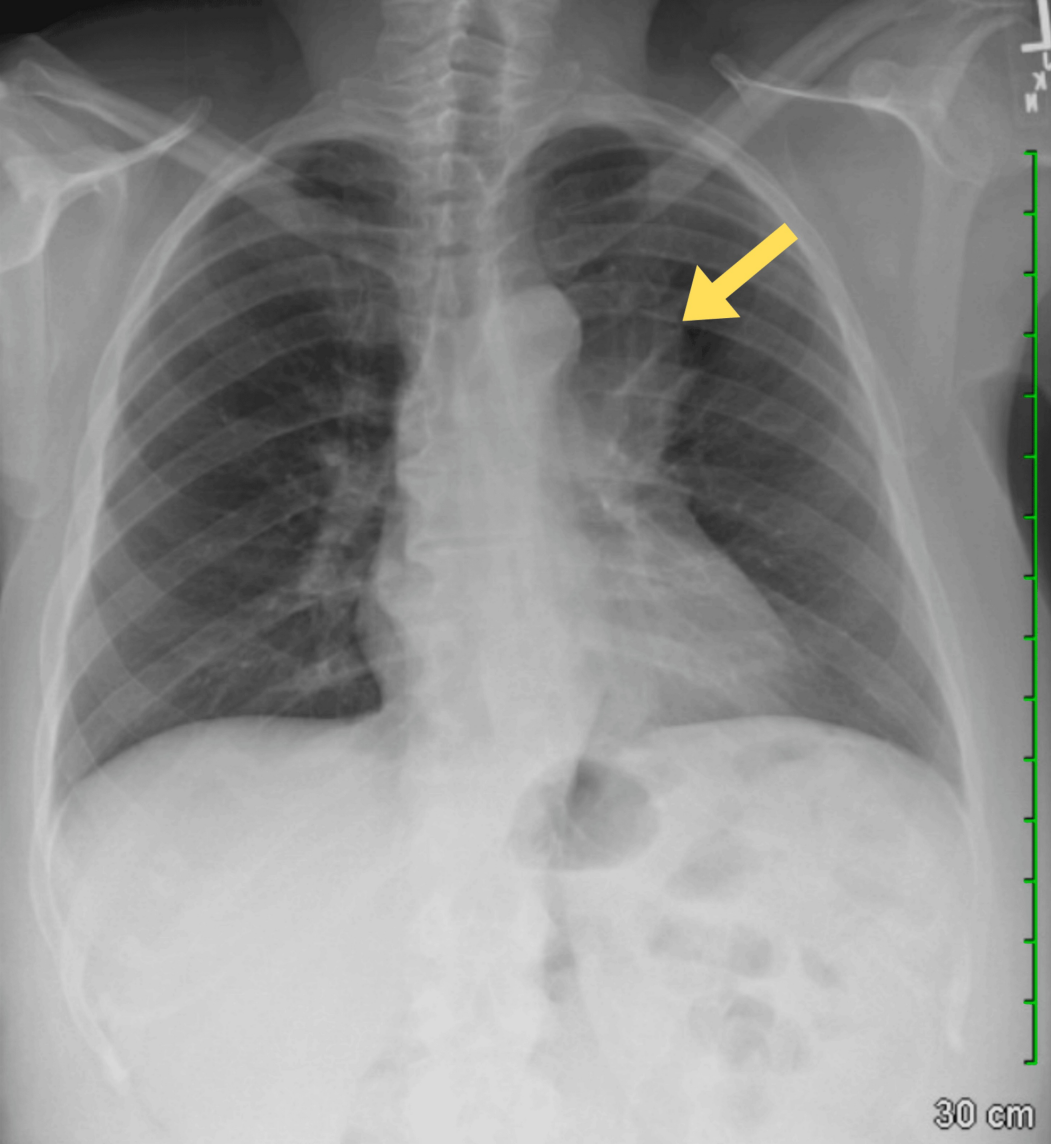
Chest X-ray showing a mediastinal mass (yellow arrow) present at the level of the aortic arch and aortopulmonary window. Bony structures display moderate degenerative changes.

The patient was initially treated with medications for pain management and was closely monitored for any deterioration in her condition. Following a thorough evaluation and discussion with the patient, a surgical consultation was arranged. The surgical team determined that a complete resection of the mediastinal mass was necessary to alleviate symptoms and address the underlying pathology. Preoperative imaging, including a chest X-ray, was performed and identified the mass situated at the level of the aortic arch (Figure 2).

On the day of the surgery, the patient was placed in a supine position. Hemodynamic monitoring lines and catheters were placed, and the patient was brought under general endotracheal anesthesia. The patient was then prepped and draped in a sterile fashion. A midline chest incision was performed, and dissection was carried down to the level of the sternum using electrocautery. A median sternotomy was performed, with the sternal edges made hemostatic using electrocautery and Gelfoam. The left hemisternum was elevated using the internal mammary artery (IMA) retractor, and the left pleural space was entered.

The mediastinal mass was carefully dissected from the surrounding pericardium and lung tissue, with minor air leaks occurring due to small rents in the lung, which densely adhered to the mass. The mass was removed en bloc and sent for pathology to confirm the diagnosis of thymoma. Hemostasis was achieved with electrocautery. A 36 French chest tube was placed in the left chest, and a 24 French Blake drain was positioned above the pericardium. The sternum was closed with five stainless steel wires and the wound was closed in three layers. Postoperative chest X-ray showed atelectasis at the left lung base, an enlarged cardiac silhouette, and subcutaneous air in the left chest wall.

Following the surgical resection of the thymoma, the patient was initiated on intravenous immunoglobulin (IVIG) therapy. Throughout her hospitalization, the patient's clinical status was meticulously monitored, with particular attention given to the alleviation of myasthenia gravis symptoms, such as muscle weakness and fatigue, and the prevention of infections, given her immunocompromised state. Regular laboratory tests were conducted to evaluate her immune status, including immunoglobulin levels, and to gauge the effectiveness of the IVIG therapy. After two days of IVIG treatment, the patient showed significant clinical improvement, with a marked reduction in muscle weakness and overall stabilization of her health. She experienced no further complications during her hospital stay.

## Discussion

Thymomas presenting with myasthenia gravis-related weakness and immunodeficiency, as seen in Good's syndrome, create a complex clinical picture. The overlap between these conditions secondary to thymoma is well-documented in paraneoplastic literature. In a study of 89 patients with Good's syndrome, 15.7% were also found to have myasthenia gravis [1,6]. Thymomas typically arise in the upper anterior mediastinum, where the thymus is normally located, but can occasionally develop in the posterior mediastinum or lower neck. Extrathoracic metastasis is uncommon, and thymomas often grow asymptomatically, being incidentally discovered during imaging studies. When a thymoma is suspected, initial imaging should begin with a chest X-ray, followed by a chest CT scan [7]. Complete surgical resection of the thymoma is recommended for eligible patients, though recurrence rates vary widely, ranging from 5% to 50%, with an average disease-free interval of around five years [8]. It is important to note that thymomas can be associated with various paraneoplastic syndromes, including Lambert-Eaton myasthenic syndrome, myositis, encephalitis, Isaac's syndrome, and Morvan's syndrome [2].

When evaluating a middle-aged patient presenting with chest pain, dyspnea, and weakness, the differential diagnosis should be broad due to the nonspecific nature of these symptoms. Potential diagnoses may include acute coronary syndrome (ACS), pneumonia, and chronic heart failure exacerbation, among others. However, once a mediastinal mass is identified, the differential diagnosis narrows significantly to include thymoma and other mediastinal tumors. Radiographic imaging, such as a chest X-ray, can be pivotal in identifying abnormalities that may suggest a malignancy in the anterior mediastinal region, which could explain the chest pain and dyspnea [7]. Weakness is another nonspecific symptom that can only be appropriately contextualized once an anterior mediastinal mass is identified, suggesting a possible etiology such as myasthenia gravis. Confirmatory testing, including antibody assays and EMG, can then be utilized to diagnose myasthenia gravis [2]. Given the rarity of concurrent thymoma and Good's syndrome, the importance of maintaining a high index of suspicion in patients with such symptoms cannot be overstated. In cases where a thymoma has been previously diagnosed and new myasthenia gravis-like symptoms arise, a CT scan should be the first-line imaging modality. The definitive diagnosis of a suspected thymoma is achieved through a combination of cytopathology and histopathology [8]. Our patient underwent a comprehensive diagnostic process, including initial chest X-ray imaging that led to the discovery of a mass, followed by chest CT for better characterization of the findings, myasthenia gravis-specific testing to thoroughly investigate the symptoms, and finally, an incisional biopsy to confirm the diagnosis.

Developing a management plan for thymoma is multifaceted and involves several key considerations. Ideally, complete surgical resection is preferred if the tumor is localized, the patient is hemodynamically stable, and symptoms related to myasthenia gravis are well-controlled [2]. Treatment options for myasthenia gravis include corticosteroids, other immunosuppressants or immunomodulators, IVIG, or plasmapheresis, which removes circulating antibodies that cause neuromuscular weakness [2,9]. The choice of treatment for myasthenia gravis can vary depending on the specific antibodies present. For myasthenia gravis patients testing positive for acetylcholine receptor antibodies, a thymectomy may be indicated, as this procedure can improve symptoms. However, for patients who test positive only for muscle-specific tyrosine kinase (MuSK) antibodies, thymectomy may not be recommended.

Clinicians need to carefully weigh the risks and benefits of thymectomy in the context of the patient's overall health, particularly in those with Good's syndrome, given the increased risk of postoperative complications due to their immunodeficient state. It is crucial to stabilize the patient's neuromuscular symptoms before considering thymectomy, as the stress of the surgery could exacerbate respiratory failure [2,9]. In cases where the thymoma is not resectable, chemotherapy may be considered as an alternative treatment option. Previous literature has suggested using a combination of cisplatin and anthracycline, though there is no universally accepted chemotherapy regimen for thymoma. Even for recurrent thymomas, surgery remains the first-line treatment when feasible [2]. Long-term follow-up and monitoring are essential, particularly in patients with Good's syndrome, as their immunodeficiency requires ongoing management to prevent infections and other complications.

Good's syndrome is a less common manifestation associated with thymoma, with an estimated incidence ranging from 6% to 11% among patients with thymomas [4]. The most common clinical presentation in patients with Good's syndrome is infection [10]. Sinopulmonary infections are the most common, followed by urinary tract infections (UTIs) and skin infections [4]. Bloodwork in Good's syndrome patients typically reveals low serum immunoglobulin levels, indicating an immunodeficient state that predisposes them to opportunistic infections. While these patients are not generally at increased risk for systemic fungal infections, they may experience local mucocutaneous candidiasis [10]. *Haemophilus influenzae* and *Streptococcus pneumoniae* are two of the most common bacterial pathogens found in patients with Good's syndrome. Recent global antimicrobial susceptibility data suggest that *Streptococcus pneumoniae* is highly susceptible to vancomycin, linezolid, tigecycline, and levofloxacin, whereas *Haemophilus influenzae* shows lower susceptibility to ampicillin [10]. Additionally, about 40% of Good's syndrome patients may experience viral infections, with cytomegalovirus (CMV) being the most common [4]. The presence of Good's syndrome complicates the management of thymoma, particularly when considering thymectomy, as the immunodeficient state may worsen post-surgery, increasing the risk of infections. Moreover, if immunosuppressive therapy is used to manage myasthenia gravis in these patients, it can lead to severe consequences due to the already compromised immune system. Therefore, a multidisciplinary approach involving immunologists, oncologists, and thoracic surgeons is recommended to optimize outcomes in these complex cases. It is recommended that patients with thymoma and myasthenia gravis be screened for Good's syndrome to prevent complications [5]. The treatment of Good's syndrome typically involves addressing bacterial infections with appropriate antimicrobials and correcting immunodeficiency through immunoglobulin replacement therapy [11].

This case offers valuable insights into the rare coexistence of myasthenia gravis and Good's syndrome in the presence of a thymoma. The patient's condition remained stable, largely due to the prompt and precise surgical intervention following diagnosis, along with regular monitoring of blood work to assess the immunosuppressed state. When evaluating thymoma, it is crucial for diagnosticians to consider the broad spectrum of potential clinical manifestations and associated paraneoplastic syndromes. Myasthenia gravis is the most common paraneoplastic syndrome associated with thymoma, but patients may also present with symptoms related to anterior mediastinal compression, such as chest pain and dyspnea, as seen in this case, or even superior vena cava compression syndrome [1,2]. Awareness of the appropriate imaging techniques, such as chest X-ray and CT scans, is essential for the accurate evaluation and confirmation of thymoma [7,8]. Additionally, clinicians should recognize that the treatment strategy for thymoma must be tailored to the specific paraneoplastic syndromes present, and in cases where Good's syndrome coexists, consideration of the immunodeficient state is critical [2,5,11]. Long-term follow-up and individualized monitoring plans are crucial for detecting recurrence and managing ongoing immunodeficiency. This case highlights the importance of a thorough diagnostic approach with careful management to ensure optimal outcomes for patients with such complex presentations.

## Conclusions

This case highlights the rare occurrence of concurrent myasthenia gravis and Good's syndrome in association with a thymoma. The patient's successful outcome following prompt surgical resection and IVIG therapy demonstrates the importance of timely intervention and careful monitoring of immunological status. Clinicians should be vigilant in recognizing the broad spectrum of symptoms and potential paraneoplastic syndromes associated with thymomas, including myasthenia gravis and Good's syndrome. This case emphasizes the need for appropriate diagnostic imaging and testing to accurately identify these conditions. The complexity of such cases necessitates multidisciplinary collaboration, where multiple specialties may be involved in both diagnosis and treatment, to ensure optimal patient outcomes. Additionally, given the potential for recurrence, long-term follow-up is essential for monitoring the patient’s condition and managing any future complications. Management strategies must be tailored to the specific paraneoplastic syndromes present and consider the patient's overall immunological state, particularly in cases involving Good's syndrome.
